# Supplementary material for: Liquid biopsy diagnostics for non-small cell lung cancer via elucidation of tRNA signatures
Source: Commun Med (Lond). 2025 Aug 21;5:364. doi: 10.1038/s43856-025-01068-2 (PMC12370967; doi:10.1038/s43856-025-01068-2)
Supplement: Supplementary file 2 — Supplementary Information [file 43856_2025_1068_MOESM2_ESM.pdf]

Figure S1. Transmission Electron Microscopy (TEM) Images of Exosomes Isolated from Plasma Samples
TEM images show extracellular vesicles (EVs), with arrows indicating exosomes ranging from 30 to 150 nm in diameter. The scale bar represents 100 nm.

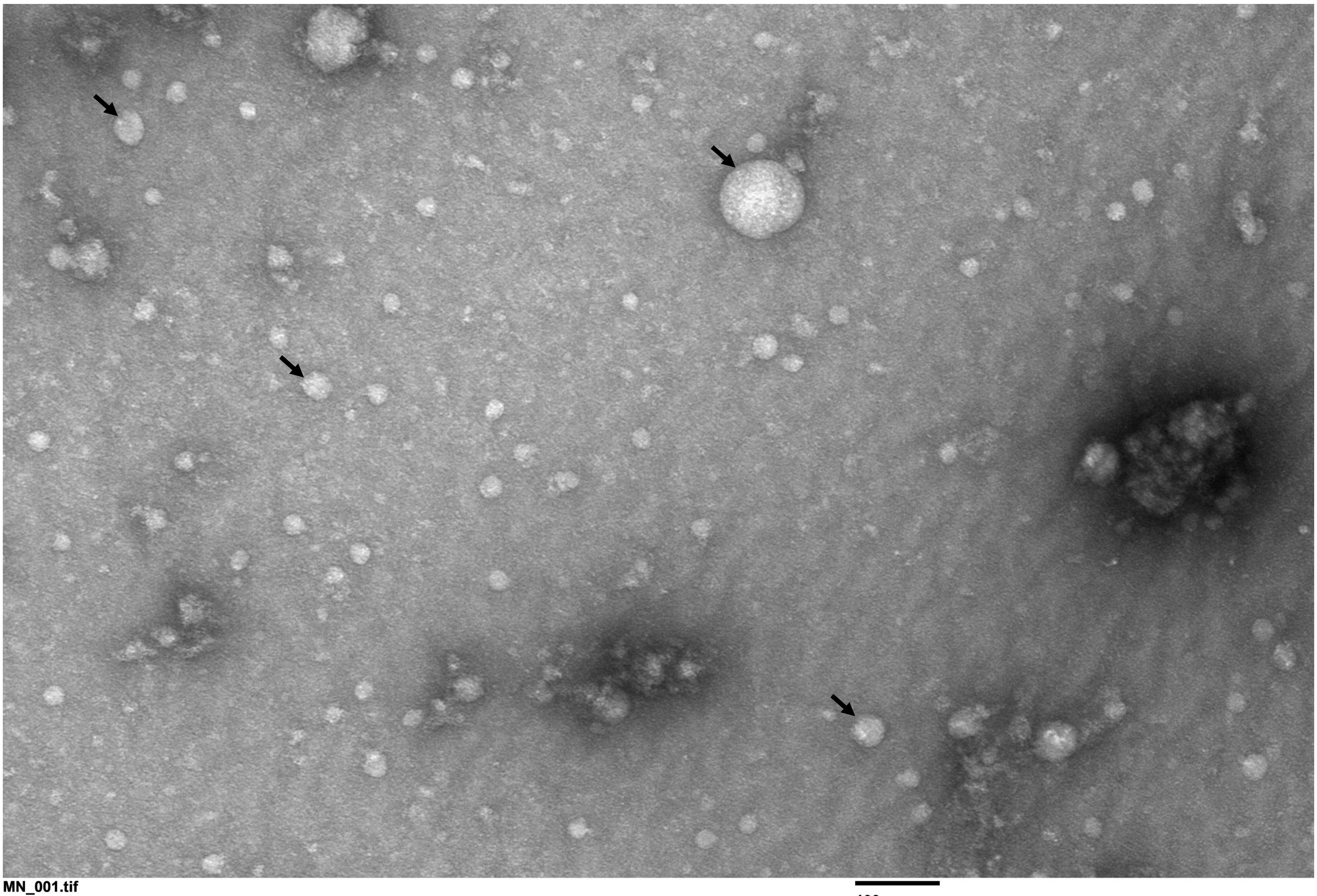

MN_001.tif

Cal: 0.315978 nm/pix
15:26 2023-12-04
TEM Mode: Imaging
Camera: BIOSPR16, Exposure: 320 (ms) x 10 std. frames, Gain: 5, Bin: 1
Gamma: 1.00, No Sharpening, Normal Contrast

100 nm
HV=100kV
Direct Mag: 50000 x
BEMF Univ of Hawaii

Figure S2. Principal Component Analysis (PCA) of tRNA Expression Data across NSCLC Subtypes and Control Group

Each point represents an individual sample, with colors indicating the respective subtype. The "Control" group includes all healthy and benign samples, serving as a baseline for comparison. The "Other" group comprises samples with unspecified NSCLC subtype information or NSCLC subtypes other than LUAD and LUSC.

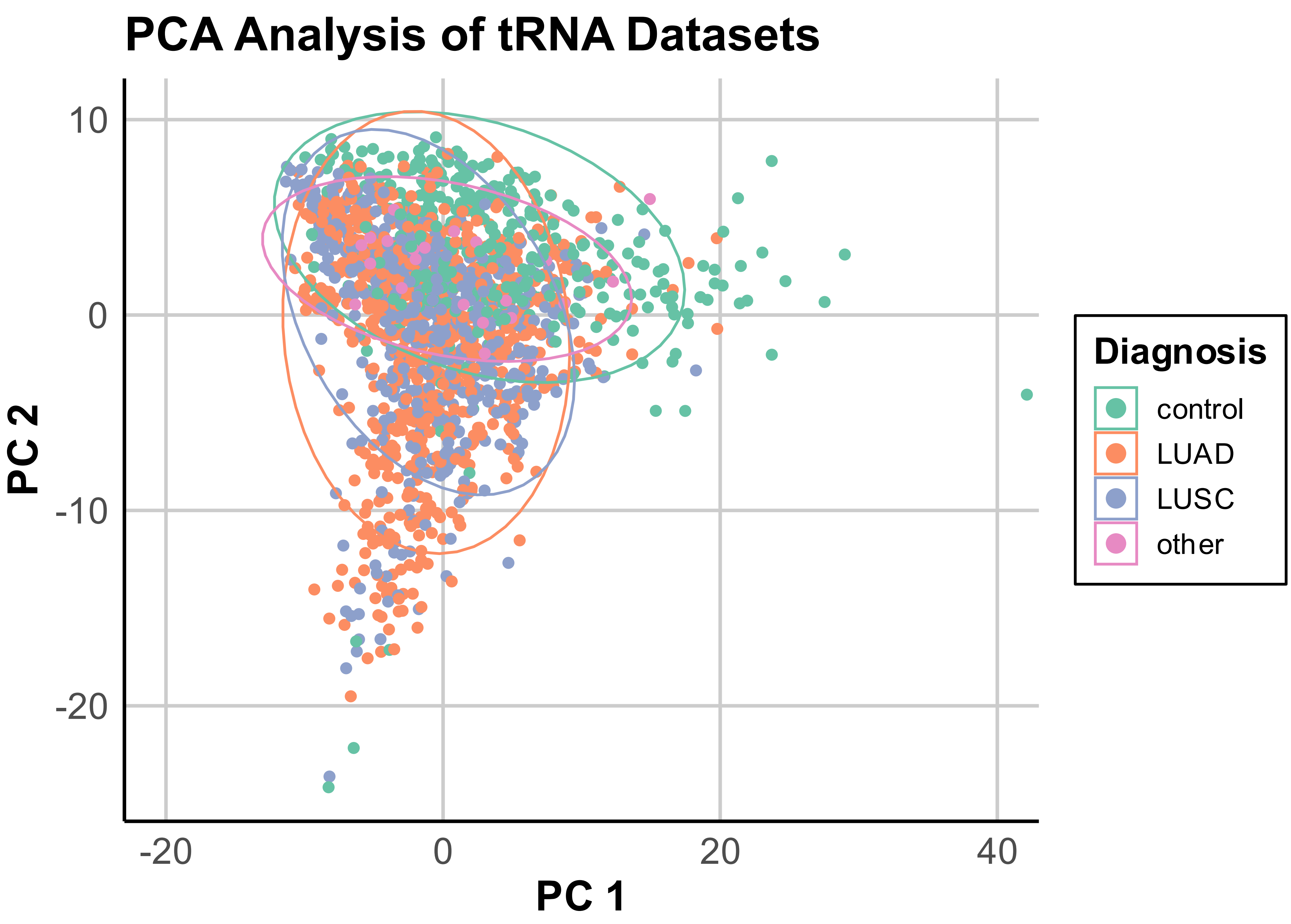

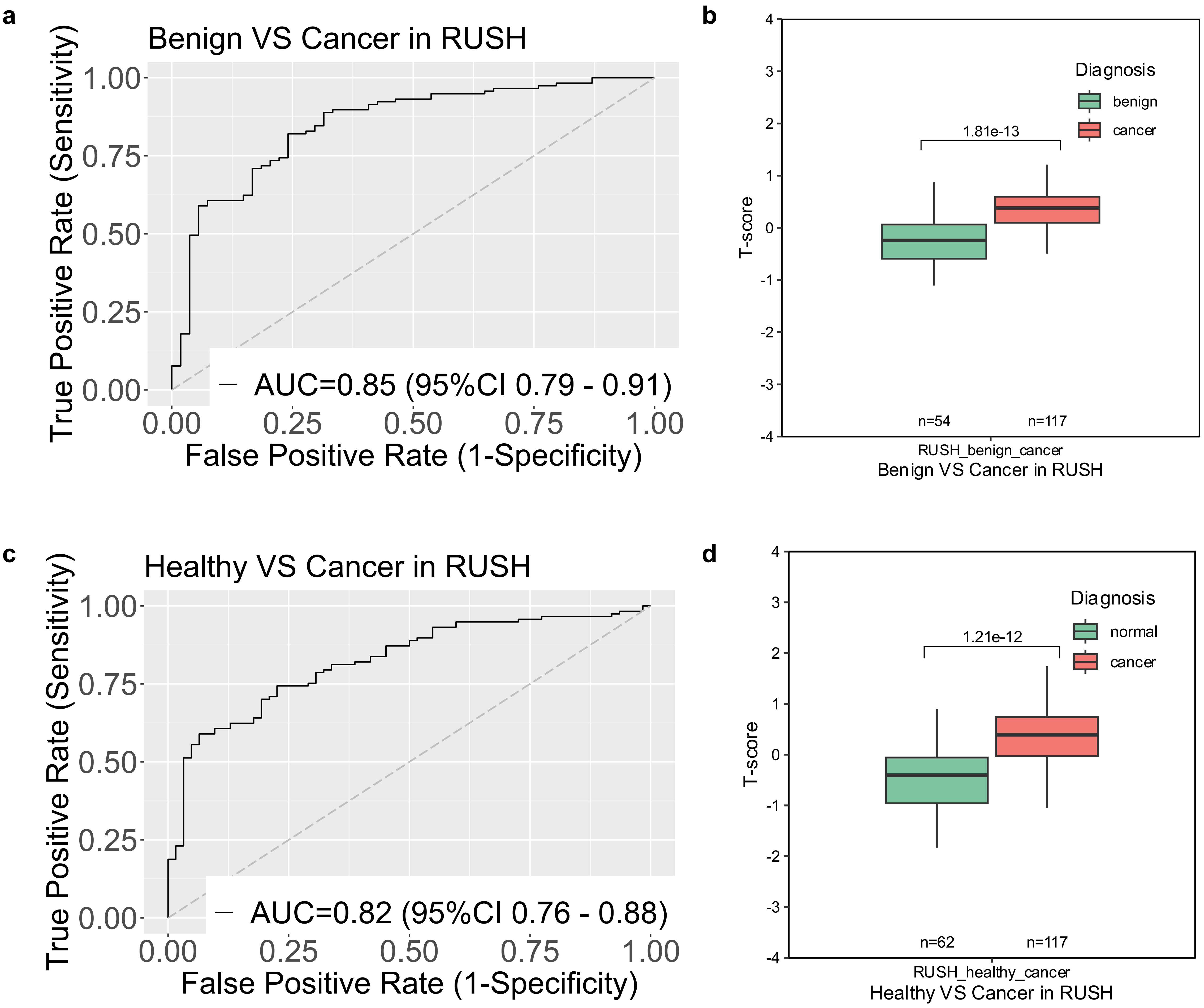

Figure S3. Model Performance and Robustness of the 6-tRNA Signature in Distinguishing Benign vs. Cancer and Healthy vs. Cancer Groups

a, c. Receiver Operating Characteristic (ROC) curves evaluating the model's diagnostic accuracy across datasets. AUC, area under the curve; CI, confidence interval.

b, d. Boxplots comparing T-scores between tumor and control groups. Box colors represent diagnostic categories. Sample sizes (n) for each cohort are displayed below the corresponding boxplots. Statistical comparisons were performed using the Mann–Whitney U test. Exact p-values are displayed above each comparison bar.

Figure S4. Receiver Operating Characteristic (ROC) Curves of Stratified and Balanced TCGA Cohorts for Comprehensive Assessment of Signature Discrimination
Curve colors correspond to their respective balanced cohorts. AUC, area under the curve; CI, confidence interval.

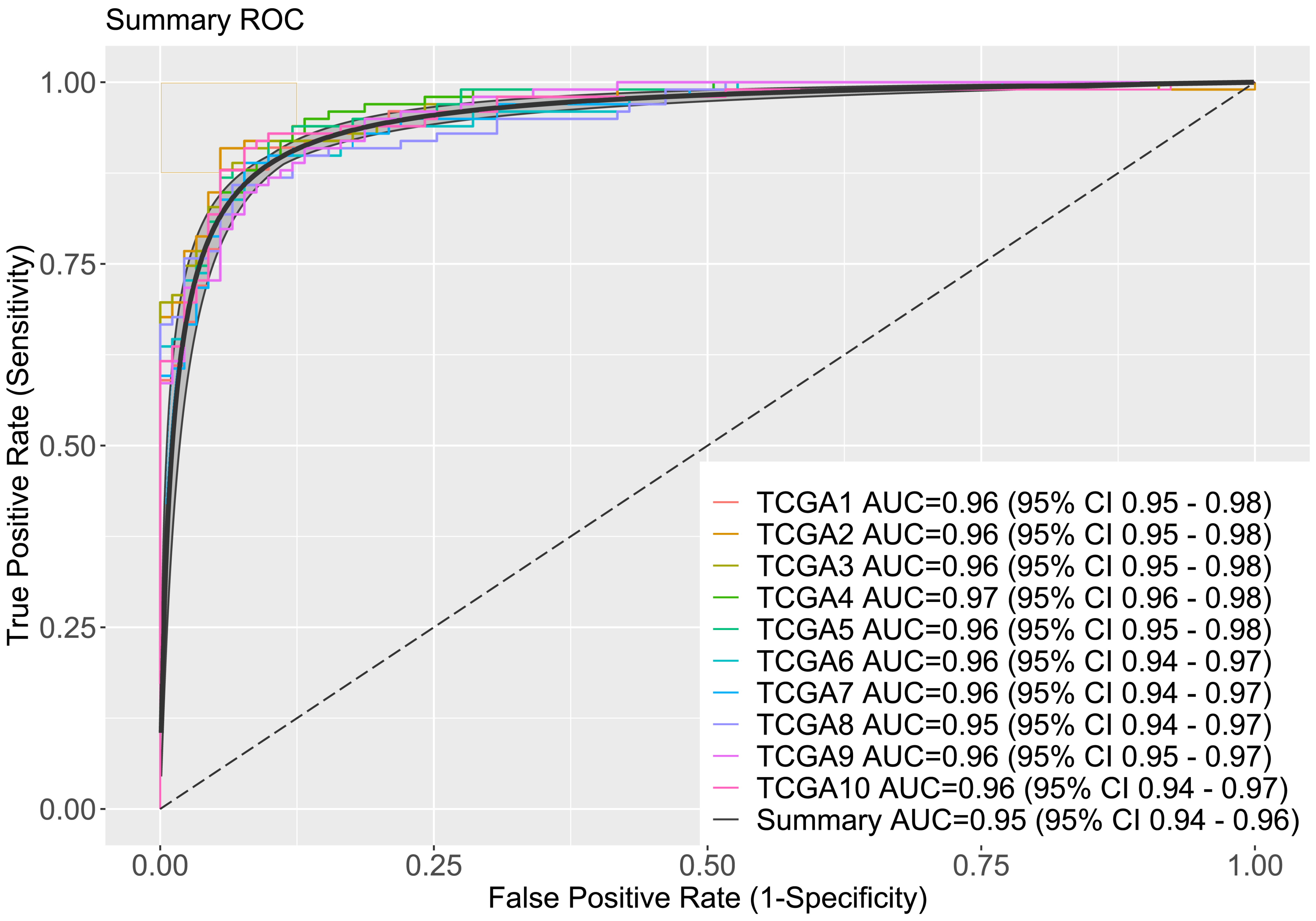

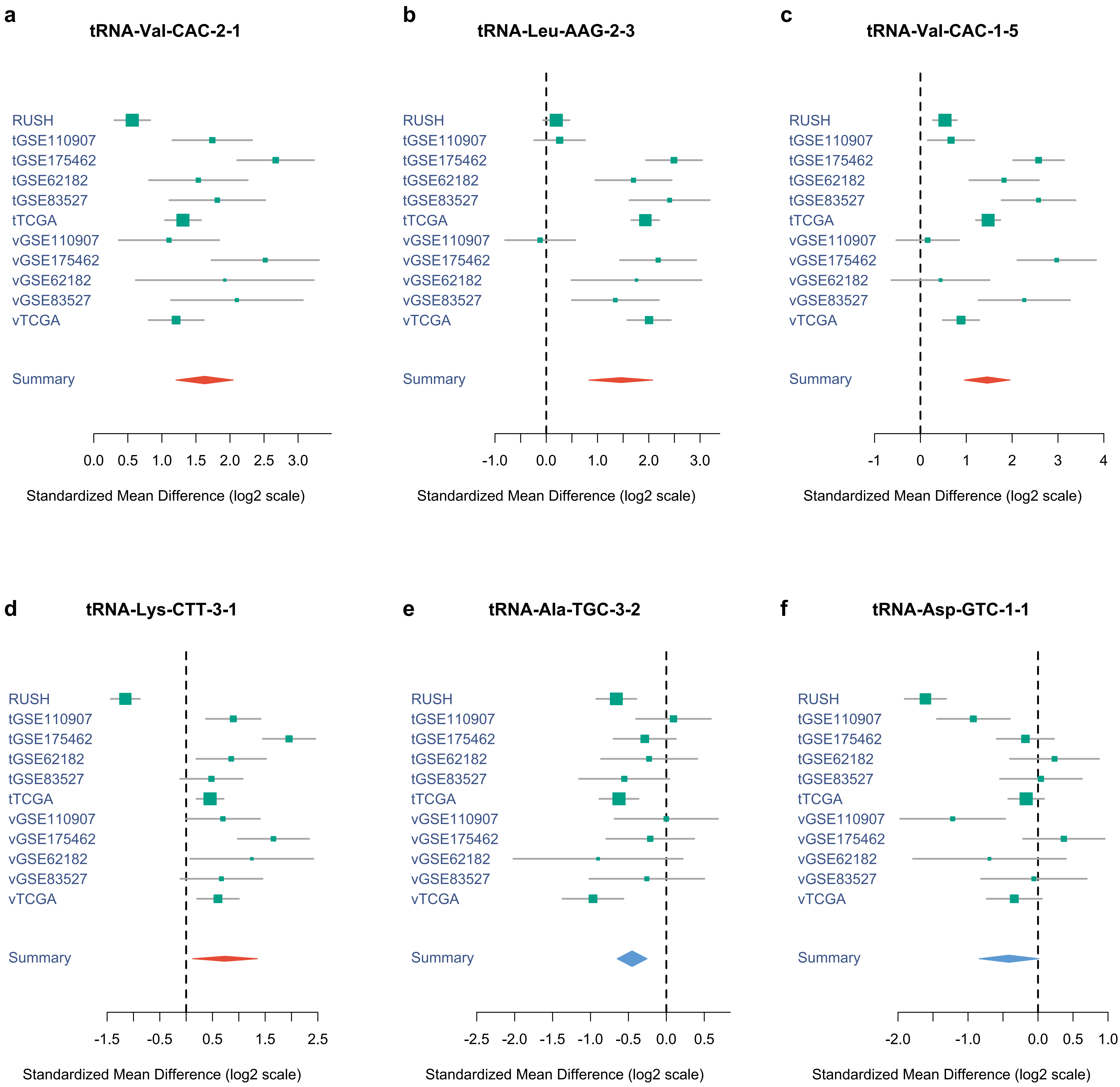

Figure S5. Meta-Analysis of Standardized Mean Differences (SMDs) for T-Scores of the 6-tRNA Signature Across Datasets

Forest plots show the meta-analysis of SMDs for T-scores in the discovery, hold-out validation, and independent validation phases. Each dataset is represented by an effect estimate (green box) with 95% confidence intervals (grey lines). Box size reflects sample size. Initial “t”, discovery phase; Initial “v”, hold-out validation phase; “RUSH”, independent validation phase. Diamonds represent overall summary estimates, where red indicates higher expression in tumors and blue indicates lower expression.

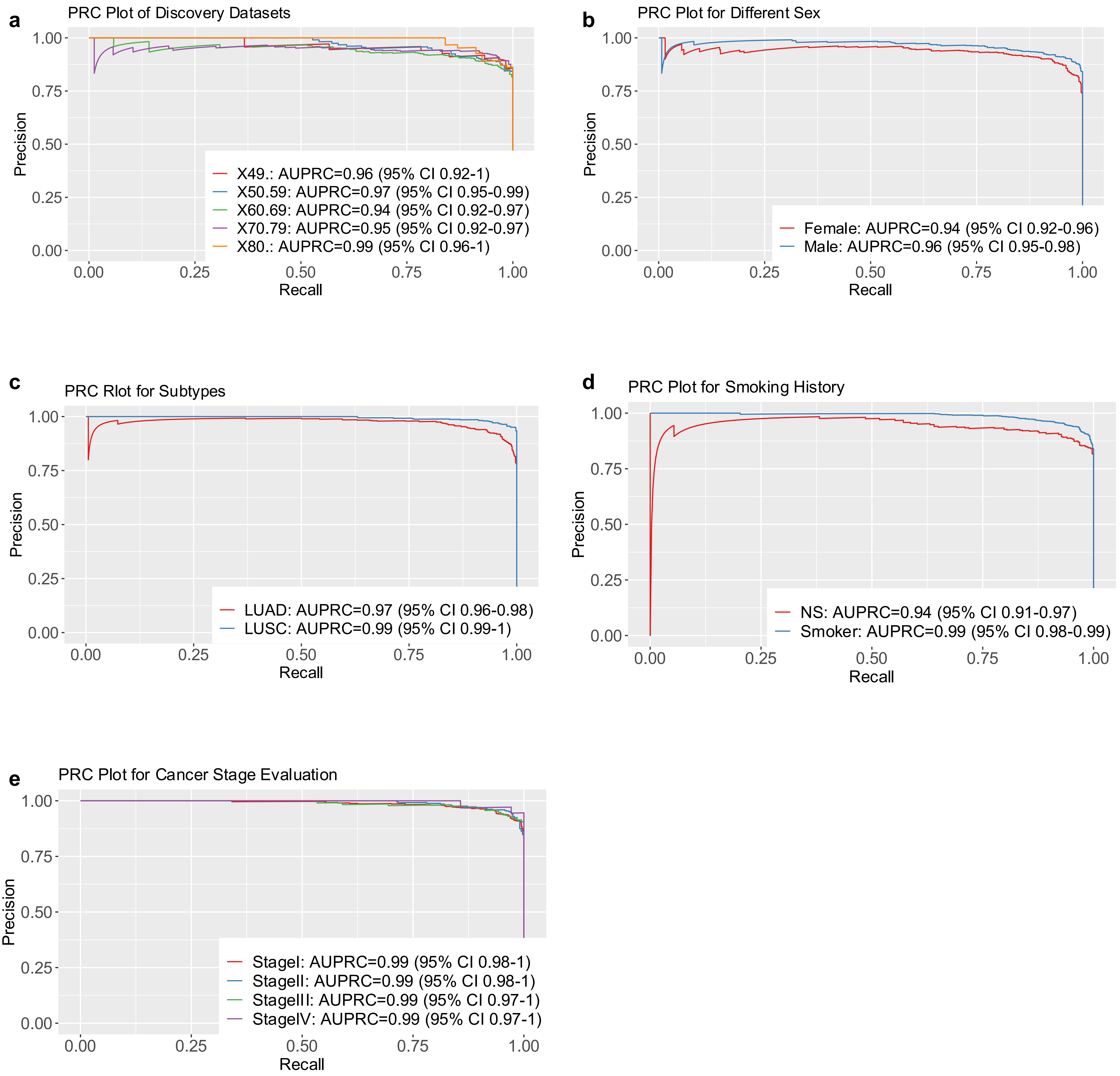

Figure S6. Precision-Recall Curve Analysis for the T-score of the 6-tRNA Signature Across Demographic and Clinical Subgroups

Precision-recall curves assess the diagnostic performance of the 6-tRNA signature T-score across various subgroups, including age, sex, histological subtype, smoking status, and AJCC pathologic stage. Curve colors indicate the respective datasets. AUPRC, area under the precision-recall curve; CI, confidence interval.

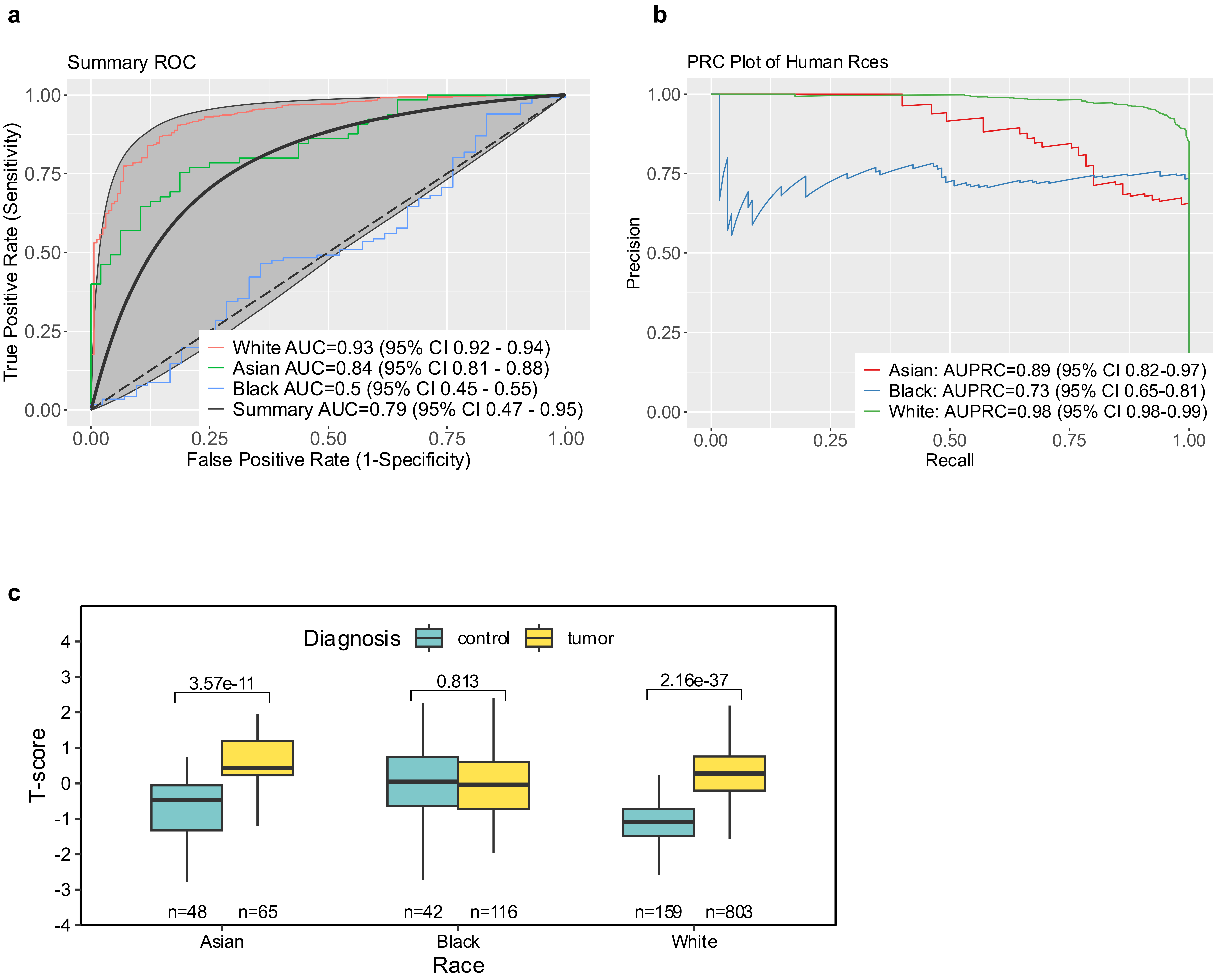

Figure S7. Diagnostic Performance of the tRNA Signature Across Different Racial Groups

a, b. Receiver Operating Characteristic (ROC) curves and Precision-Recall (PRC) curves evaluating the diagnostic accuracy of the T-score of the 6-tRNA signature across racial groups. Curve colors correspond to respective datasets. AUC, area under the curve; AUPRC, area under the precision-recall curve; CI, confidence interval.

c. Boxplots comparing T-scores between tumor and control groups across racial groups. Box colors represent diagnostic categories. Sample sizes (n) for each cohort are displayed below the corresponding boxplots. Statistical comparisons were performed using the Mann–Whitney U test. Exact p-values are displayed above each comparison bar.

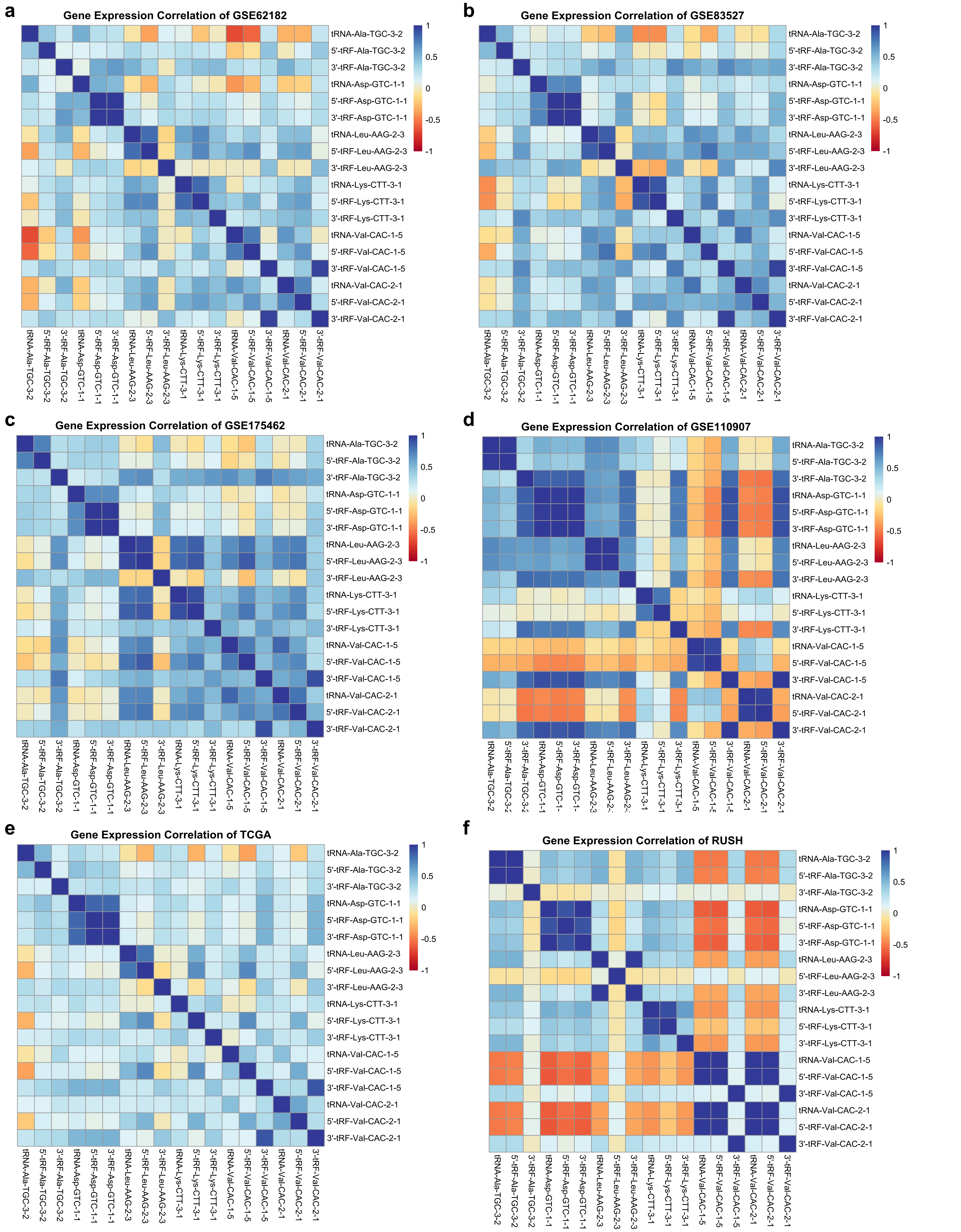

Figure S8. Spearman Correlation Heatmaps of Signature tRNAs and Corresponding tRFs Across Datasets
Heatmaps displaying Spearman's rank correlation coefficients between signature tRNAs and their corresponding tRFs across different datasets. The color scale represents correlation values, ranging from -1 (red) to 1 (dark blue).

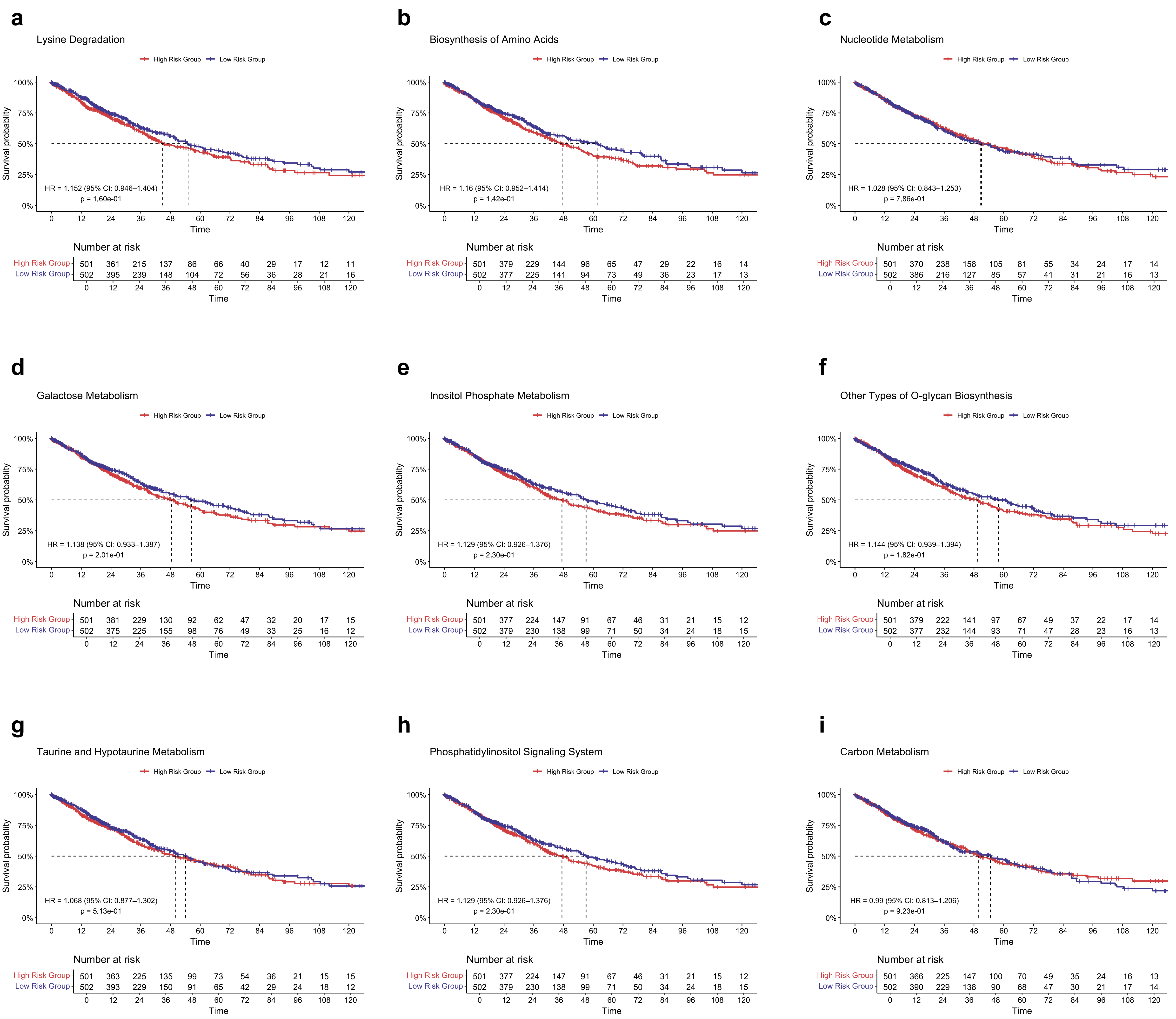

Figure S9. Kaplan-Meier Survival Analysis of Non-Significant Metabolic Pathways
Kaplan-Meier survival curves comparing high-risk and low-risk groups based on pathway activity scores. Dashed lines indicate the median survival threshold (50% survival probability) at specific time points. The tables below the plots display the number of patients at risk over the observation period. Statistical significance was assessed using the log-rank test, with no significant differences observed. HR, hazard ratio; CI, confidence interval.
