# Supplementary material for: Liquid biopsy diagnostics for non-small cell lung cancer via elucidation of tRNA signatures
Source: Commun Med (Lond). 2025 Aug 21;5:364. doi: 10.1038/s43856-025-01068-2 (PMC12370967; doi:10.1038/s43856-025-01068-2)
Supplement: Supplementary file 3 — Description of Additional Supplementary files [file 43856_2025_1068_MOESM3_ESM.pdf]

Description of Additional Supplementary files

File name: Supplementary Data 1

Description: Metadata of all samples from public datasets.

File name: Supplementary Data 2

Description: Initial filtering of tRNAs based on statistical significance and effect size.

File name: Supplementary Data 3

Description: List of tRFs filtered based on statistical significance and effect size.

File name: Supplementary Data 4

Description: Prediction results of hybrid genes interacting with targeted tRFs.

File name: Supplementary Data 5

Description: KEGG pathway enrichment analysis results of tRF-targeted Genes.

File name: Supplementary Data 6

Description: GO enrichment analysis results of tRF-targeted Genes.

File name: Supplementary Data 7

Description: KEGG analysis results of tRF-targeted genes in metabolic pathways.
