# Supplementary material for: Liquid biopsy diagnostics for non-small cell lung cancer via elucidation of tRNA signatures
Source: Commun Med (Lond). 2025 Aug 21;5:364. doi: 10.1038/s43856-025-01068-2 (PMC12370967; doi:10.1038/s43856-025-01068-2)
Supplement: Supplementary file 11 — Reporting Summary [file 43856_2025_1068_MOESM11_ESM.pdf]

nature portfolio

Corresponding author(s): Youping Deng; Yuanyuan Fu; Lang Wu

Last updated by author(s): Jul 22, 2025

Reporting Summary

Nature Portfolio wishes to improve the reproducibility of the work that we publish. This form provides structure for consistency and transparency in reporting. For further information on Nature Portfolio policies, see our Editorial Policies and the Editorial Policy Checklist.

Statistics

For all statistical analyses, confirm that the following items are present in the figure legend, table legend, main text, or Methods section.

n/a	Confirmed	
☐	☒	The exact sample size (*n*) for each experimental group/condition, given as a discrete number and unit of measurement
☒	☐	A statement on whether measurements were taken from distinct samples or whether the same sample was measured repeatedly
☐	☒	The statistical test(s) used AND whether they are one- or two-sided<br>*Only common tests should be described solely by name; describe more complex techniques in the Methods section.*
☐	☒	A description of all covariates tested
☐	☒	A description of any assumptions or corrections, such as tests of normality and adjustment for multiple comparisons
☐	☒	A full description of the statistical parameters including central tendency (e.g. means) or other basic estimates (e.g. regression coefficient) AND variation (e.g. standard deviation) or associated estimates of uncertainty (e.g. confidence intervals)
☐	☒	For null hypothesis testing, the test statistic (e.g. *F*, *t*, *r*) with confidence intervals, effect sizes, degrees of freedom and *P* value noted<br>*Give P values as exact values whenever suitable.*
☒	☐	For Bayesian analysis, information on the choice of priors and Markov chain Monte Carlo settings
☒	☐	For hierarchical and complex designs, identification of the appropriate level for tests and full reporting of outcomes
☐	☒	Estimates of effect sizes (e.g. Cohen's *d*, Pearson's *r*), indicating how they were calculated

Our web collection on statistics for biologists contains articles on many of the points above.

Software and code

Policy information about availability of computer code

Data collection	Sources of data included raw sequencing files (FASTQ format) of lung tissue samples retrieved from the GEO database (datasets GSE110907, GSE62182, GSE83527, GSE175462) and the TCGA database (datasets TCGA-LUAD, TCGA-LUSC). Associated demographic and clinical information, such as sex, age, histological subtype, AJCC pathological stage, and smoking history, were also obtained from these databases. Additionally, plasma specimens and patient information were collected from RUSH University Medical Center. Exosomal RNA was extracted from these plasma samples, followed by small RNA sequencing to obtain raw sequencing files (FASTQ format). No custom software was used for data collection. The focus was on retrieving and organizing data from established databases and approved institutional sources.
Data analysis	All small RNA sequencing raw FASTQ files underwent quality control using FastQC (version 0.11.9) for initial assessment and fastp (version 0.20.0) for adaptor trimming. The processed reads were aligned with STAR software (version 2.7.10), and gene annotation was performed using FeatureCounts (version 2.0.6). For downstream analysis, we adopted MetaIntegrator (version 2.1.3) to identify a six-tRNA diagnostic signature, validating via leave-one-out cross-validation, forwardSearch/backwardSearch optimization, and ROC/PRC analysis. Kaplan-Meier survival analysis and multivariate Cox regression evaluated prognostic relevance, while Spearman correlation examined tRF-tRNA relationships. Functional analysis was conducted using RNAhybrid (version 2.1.2), KEGG (https://david.ncifcrf.gov), and GO enrichment (https://www.geneontology.org/), ensuring robustness and reproducibility. The results were visualized via ggplot2 package in R and Cytoscape (version 3.10.1). All analyses were conducted in accordance with the package's guidelines and its open-source licensing terms. The primary code is available at https://github.com/F-UH/Primary-Code/tree/master.

For manuscripts utilizing custom algorithms or software that are central to the research but not yet described in published literature, software must be made available to editors and reviewers. We strongly encourage code deposition in a community repository (e.g. GitHub). See the Nature Portfolio guidelines for submitting code & software for further information.

Data

Policy information about availability of data

All manuscripts must include a data availability statement. This statement should provide the following information, where applicable:

- Accession codes, unique identifiers, or web links for publicly available datasets
- A description of any restrictions on data availability
- For clinical datasets or third party data, please ensure that the statement adheres to our policy

Small RNA-seq data and associated clinical information were retrieved from the Gene Expression Omnibus (GEO) database under the following accession numbers: GSE110907, GSE62182, GSE83527, and GSE175462. These datasets can be accessed through the respective URLs:
• GSE110907: https://www.ncbi.nlm.nih.gov/geo/query/acc.cgi?acc=GSE110907;
• GSE62182: https://www.ncbi.nlm.nih.gov/geo/query/acc.cgi?acc=GSE62182;
• GSE83527: https://www.ncbi.nlm.nih.gov/geo/query/acc.cgi?acc=GSE83527;
• GSE175462: https://www.ncbi.nlm.nih.gov/geo/query/acc.cgi?acc=GSE175462;
Data from lung cancer cohorts, including TCGA-LUAD and TCGA-LUSC, were retrieved from The Cancer Genome Atlas (TCGA) database. These datasets are publicly available at the following URL: https://portal.gdc.cancer.gov/analysis_page?app= .
The small RNA sequencing data from the RUSH cohort have been deposited in the database of Genotypes and Phenotypes (dbGaP) under accession number phs004166.v1.p1. The data will be made available to authorized researchers upon controlled access approval.

Research involving human participants, their data, or biological material

Policy information about studies with human participants or human data. See also policy information about sex, gender (identity/presentation), and sexual orientation and race, ethnicity and racism.

Reporting on sex and gender	The study incorporates sex as one of the control variables from demographic factors. A total of 821 females and 858 males were included in the analysis. Given the biological differences between sexes, we validated the identified signature separately for males and females to ensure its general applicability. Additionally, a multivariate Cox regression analysis was conducted to analyze the risk score, with sex included as one of the variables. The results indicated that sex does not significantly impact the predictive accuracy of the risk score for non-small cell lung cancer.
Reporting on race, ethnicity, or other socially relevant groupings	In our study, race was incorporated as a control variable within demographic factors. The racial groups included were White (n=962), African American (n=158), and Asian (n=113). These categories were based on self-reported demographic data collected as part of the dataset. Given the potential biological and genetic differences among racial groups, we validated the identified diagnostic signature separately for each racial category to assess its diagnostic generality. The results demonstrated that our signature effectively diagnosed lung cancer in White and Asian populations but was less robust for African Americans. This may be attributed to the relatively smaller sample size of African American participants, which could impact the statistical power of our analysis.
Population characteristics	The study also considered other population characteristics, such as age, histological subtype, AJCC pathological stage, and smoking history of the participants (detailed information is provided in Table 1 of the manuscript). Similar to sex, these factors were evaluated during the signature validation and included in the Cox regression analysis to assess the risk score.
Recruitment	The publicly available data were obtained from the TCGA and GEO datasets as previously mentioned. Plasma specimens and associated patient information were purchased from RUSH University Medical Center, received ethical approval from the University of Hawaii Human Studies Program under protocol number 2018-00636.
Ethics oversight	The acquisition and utilization of human blood samples, along with the clinical data, received ethical approval from the University of Hawaii Human Studies Program under protocol number 2018-00636.

Note that full information on the approval of the study protocol must also be provided in the manuscript.

Field-specific reporting

Please select the one below that is the best fit for your research. If you are not sure, read the appropriate sections before making your selection.

☒ Life sciences ☐ Behavioural & social sciences ☐ Ecological, evolutionary & environmental sciences

For a reference copy of the document with all sections, see nature.com/documents/nr-reporting-summary-flat.pdf

Life sciences study design

All studies must disclose on these points even when the disclosure is negative.

Sample size	A comprehensive analysis was conducted on a substantial dataset comprising 1679 samples (Table S1), including 1446 tissue samples sourced from six distinct public datasets (GSE110907, GSE62182, GSE83527, GSE175462, TCGA-LUAD, and TCGA-LUSC), as well as 233 exosome samples obtained from RUSH University.
Data exclusions	Samples exhibiting low expression in more than half of the tRNAs were excluded. This criterion ensured the inclusion of only high-quality samples with adequate expression levels for reliable analysis.

Replication	The study focuses on analyzing RNA sequencing data from human tissue and plasma samples to identify diagnostic signatures for non-small cell lung cancer (NSCLC). For the tissue samples, we collected the sequencing data of 1,173 cancer samples and 273 non-cancer samples. In the case of plasma samples, we sequenced 117 cancer samples, 62 normal samples, and 54 benign samples.
Randomization	Initially, samples from the public database were randomly allocated, with 70% designated for the discovery phase to facilitate model training and the remaining 30% reserved for the hold-out validation phase to assess model performance. Subsequently, all plasma samples were utilized for independent validation.
Blinding	The researchers conducting the data analysis were blinded to the clinical information of the samples, ensuring that the identification of diagnostic signatures and subsequent analyses were performed without any preconceived biases related to the participants' demographic or clinical characteristics. This approach was applied consistently throughout the model training, validation, and independent validation phases to maintain the integrity and objectivity of the study's findings.

Reporting for specific materials, systems and methods

We require information from authors about some types of materials, experimental systems and methods used in many studies. Here, indicate whether each material, system or method listed is relevant to your study. If you are not sure if a list item applies to your research, read the appropriate section before selecting a response.

Materials & experimental systems

n/a	Involved in the study
☒	☐ Antibodies
☒	☐ Eukaryotic cell lines
☒	☐ Palaeontology and archaeology
☒	☐ Animals and other organisms
☐	☒ Clinical data
☒	☐ Dual use research of concern
☒	☐ Plants

Methods

n/a	Involved in the study
☒	☐ ChIP-seq
☒	☐ Flow cytometry
☒	☐ MRI-based neuroimaging

Clinical data

Policy information about clinical studies

All manuscripts should comply with the ICMJE guidelines for publication of clinical research and a completed CONSORT checklist must be included with all submissions.

Clinical trial registration	This study was not registered as a clinical trial, as it primarily involved the analysis of existing public data and plasma samples purchased from RUSH University Medical Center. The study focused on the identification and validation of diagnostic signatures for non-small cell lung cancer using RNA sequencing data.
Study protocol	The study protocol was approved by the University of Hawaii Human Studies Program under protocol number 2018-00636. The protocol included the collection and analysis of RNA sequencing data from human tissue and plasma samples to identify diagnostic signatures for non-small cell lung cancer.
Data collection	Data collection took place at multiple sites, including RUSH University Medical Center and various public databases. The RNA sequencing data of human tissue and plasma samples were systematically retrieved from six study cohorts: GSE110907 (Korea), GSE62182 (Canada), GSE83527 (Canada), GSE175462 (Canada) from the GEO database, and TCGA-LUAD (USA) and TCGA-LUSC (USA) from the TCGA database. Plasma specimens and associated patient information were acquired from RUSH University Medical Center. The data collection period spanned from January 2018 to December 2022. All collected data were de-identified and stored in secure databases, ensuring compliance with ethical guidelines and data protection regulations.
Outcomes	The primary outcome of this study was the identification and validation of a diagnostic signature for non-small cell lung cancer (NSCLC). The effectiveness of the diagnostic signature was assessed by its ability to differentiate between cancerous and non-cancerous samples, as well as its performance in predicting the risk score of NSCLC in different subpopulations. The secondary outcomes included the evaluation of the diagnostic signature's general applicability across various demographic factors such as age, sex, histological subtype, AJCC pathologic stage, and smoking history. These outcomes were assessed using multivariate Cox regression analysis and independent validation on plasma samples to ensure robustness and reliability. The results were documented and analyzed to determine the diagnostic signature's potential clinical utility.

Plants

Seed stocks	*Report on the source of all seed stocks or other plant material used. If applicable, state the seed stock centre and catalogue number. If plant specimens were collected from the field, describe the collection location, date and sampling procedures.*
Novel plant genotypes	*Describe the methods by which all novel plant genotypes were produced. This includes those generated by transgenic approaches, gene editing, chemical/radiation-based mutagenesis and hybridization. For transgenic lines, describe the transformation method, the number of independent lines analyzed and the generation upon which experiments were performed. For gene-edited lines, describe the editor used, the endogenous sequence targeted for editing, the targeting guide RNA sequence (if applicable) and how the editor was applied.*
Authentication	*Describe any authentication procedures for each seed stock used or novel genotype generated. Describe any experiments used to assess the effect of a mutation and, where applicable, how potential secondary effects (e.g. second site T-DNA insertions, mosiacism, off-target gene editing) were examined.*
